# Pharmacological Evaluation of the Bronchorelaxant Effect of *Waltheria indica* L. (Malvaceae) Extracts on Rat Trachea

**DOI:** 10.1155/2021/5535727

**Published:** 2021-04-26

**Authors:** Rainatou Boly, Zakaline Yabre, Mathieu Nitiema, Boubacar Yaro, Jules Yoda, Lazare Belemnaba, Sylvain Ilboudo, Noëla Hoho Estelle Youl, Innocent Pierre Guissou, Sylvin Ouedraogo

**Affiliations:** ^1^Institute of Research in Health Science (IRSS), 03 PO 7047, Ouagadougou 03, Burkina Faso; ^2^Laboratory of Drug Development, Doctoral School of Sciences and Health, University Joseph KI-ZERBO, 03 BP 7021, Ouagadougou 03, Burkina Faso; ^3^University of Saint Thomas D'Aquin, 06 PO 10212, Ouagadougou 06, Burkina Faso

## Abstract

*Waltheria indica* L. (Malvaceae) is a plant used in Burkina Faso for the treatment of various ailments including asthma. The aim of the study was to evaluate the pharmacological relaxant effect of the leafy stem extracts of *Waltheria indica* and thereby verify claim of use in treating asthma. Aqueous decoction and hydroalcoholic extracts obtained from the powdered leafy stems were screened for the presence of some phytoconstituents. The *in vitro* relaxant effect of the two extracts was evaluated on acetylcholine- (ACh 10^−5^ M) and potassium chloride- (KCl 6 × 10^−2^ M) induced contractions on rat-isolated tracheal preparations. To examine whether the potassium (K^+^) channels are involved in the relaxant effect, glibenclamide, an ATP-sensitive potassium channel inhibitor, was used. Moreover, to assess the safety of the extracts, acute oral toxicity was carried out on mice. The phytochemical screening revealed the presence of alkaloids, flavonoids, saponins, steroids, triterpenoids, tannins, and coumarins in the hydroalcoholic extract. Tannins, steroids, triterpenoids, and coumarins were not detected in the aqueous decoction. With respective EC_50_ values of 1.517 ± 0.002 mg/mL and 1.433 ± 0.001 mg/mL on ACh-and KCl-provoked contractions, the hydroalcoholic extract was found more potent in relaxing the isolated rat tracheal preparations compared to the aqueous decoction. In the presence of glibenclamide, the relaxant effect of the hydroalcoholic extract (EC_50_ = 0.191 ± 0.002 mg/mL) increased and was higher than that of the aqueous decoction. At dose of 5000 mg/kg of body weight, the extracts did not produce deaths or any significant changes in the general behavior of mice. The results suggest that different mechanisms including modulation of calcium and potassium channels, particularly the ATP-sensitive K^+^ channels, could be involved in the relaxation effect. These findings could justify the traditional use of *W. indica* in the management of asthma.

## 1. Introduction

Asthma is a heterogenous disease characterized by multiple factors involving genetic predisposition and environmental factors [1–3]. It is one of the most common chronic diseases that present different features including bronchoconstriction, airway hyperreactivity, mucus secretion, and chronic inflammation [4–6]. Asthma symptoms include episodes of wheezing, coughing, and breath shortness [7–9]. The World Health Organization (WHO) estimates that asthma affects more than 339 million persons, both children and adults, across the world regardless the level of development [2]. The prevalence of asthma varies from 1 to 18% worldwide and the disease mostly affects poor children and people among the general population [5, 9]. In fact, about 250 000 deaths from asthma occur mostly in low- and middle-income countries [2, 10]. Few studies have been dedicated to the study of asthma in Africa as reported by Adeloye et al. [10]. In their report, the number of asthma cases is estimated at 119.3 million in 2010 [10]. Asthma represents a public health problem that is underdiagnosed and undertreated, with the consequence of increasing the disease burden, mainly in least developed countries [2, 9, 10].

According to the Global Initiative of Asthma (GINA), the treatment of asthma aims to control symptoms in order to minimize risk of exacerbations [1]. Numerous compounds have been developed for the management of asthma. These compounds act to (i) reverse or prevent bronchial smooth muscle constriction and (ii) reverse or prevent airway inflammation [11]. The current approach to the treatment of asthma consists of using corticosteroids (oral and inhaled) associated with bronchodilators (short- and long-acting beta 2-agonists), leukotriene antagonists, and long-acting anticholinergics [6, 7, 9, 12]. Nevertheless, despite the fact that most current antiasthmatic drugs have proven to be effective, the need to develop or search for new ones is necessary. Indeed, the current asthma drugs are associated with the occurrence of several side effects [6, 13, 14]. For instance, the long use of inhaled corticosteroids may be accompanied by bronchitis, nasopharyngitis, and respiratory tract infection while the common adverse effects of anticholinergic drugs (beta-2-agonists) are urinary tract infection, dyspnea, headache, cough, and nausea [14]. Furthermore, these drugs are sometimes not accessible to many patients, mainly in poor countries, due to their high cost. The annual management of asthma in terms of both direct and indirect costs in Europe and United States of America is approximately estimated at €18 billion and US$13 billion, respectively [3]. According to a recent report on the economic burden of asthma, annual direct costs varied from US$150 to US$3,000 per patient [15]. In 2005, the annual treatment of a moderate asthma costed approximately US$192 in Burkina Faso [16]. Due to the several drawbacks associated with the conventional treatment of asthma, the development of novel therapeutic approaches effective at low cost and with low side effects will alleviate asthma patients.

The use of phytomedicine or traditional herbal medicines is one of these approaches. Herbal medicines are widely used worldwide due to their important health benefits combined with reduced adverse effects and toxicity [3, 17–19]. Indeed, according to the WHO, at least 80% of African populations rely on traditional herbal medicine for their primary healthcare due to its accessibility and affordability [17, 19, 20].

Several authors reported the large and successful use of herbal medicine among both adults and children suffering from asthma [3, 7, 18, 21]. However, despite these significant findings, it is noteworthy that the pharmacological validation of a number of these plant-derived medicines still needs to be demonstrated.

In an effort to provide significant pharmacological information about antiasthmatic plants, this study focused on *Waltheria indica* L. (Malvaceae) (*syn. Waltheria americana*). The common and vernacular names of *W. indica* have been intensively reported [22]. The plant is commonly named sleepy morning, monkey bush, marsh-mallow, boter bush, or velvet leaf. Depending upon the geographic region, *W. indica* has many other names. Hence, it is called “*Nallabenda*” in Telugu, “*kafafi*” in Fulani, “*hankubath*” in Hausa, “*korikodi*” in Yoruba, “*matum kevel*” in Wolof, “*yar-yamde*” in Moore, “*Mokhutesela*” in South Africa, “*güinar*” in Mexico, “*uhaloa*” in Hawaii, and “*malvavisco*” in Spanish [22]. *Waltheria indica* is a short-lived shrub reaching 2–7 m height and a stem diameter of 2 cm. The plant is widely present in subtropical and tropical regions and can grow on many areas including roadside weed, old pastures, inundated savannas, or riverbanks [23, 24]. Different parts of this plant are customarily used to treat diverse ailments including pain, cancer, inflammation, cough, sore throat, lung infections, and asthma [23, 25–27]. Ethnobotanical data gathered from traditional healers confirmed the use of the aerial parts, roots, and whole plant of *Waltheria indica* to treat asthma in Burkina Faso [16]. Preclinical studies indicated that *Waltheria indica* and its active compounds exhibited analgesic, anti-inflammatory, cancer chemopreventive effect, antioxidant, antidiabetic, antiviral, antibacterial, bronchorelaxant, and other potential pharmacological activities [23, 25, 28–31]. The pharmacological effect of *W. indica* is based on the presence of numerous bioactive compounds such as tannins, flavonoids, terpenes, alkaloids, carbohydrates, sterols, cardiac glycosides, and anthraquinones [22].

Few reports have been dedicated to the study of the use of *Waltheria indica* L. for the management or treatment of asthma. Therefore, the present study was undertaken to investigate the pharmacological relaxant effect of the leafy stem extracts of *Waltheria indica* on rat trachea. Furthermore, this study aims to provide an attempt to explain the mechanism of the bronchorelaxant action.

## 2. Materials and Methods

### 2.1. Chemicals and Reagents

All chemicals and reagents used to carry out the experiments were of analytical grade. Acetylcholine was purchased from Sigma-Aldrich (St. Louis, MO). Potassium chloride (KCl) was supplied by Labosi (France). Tablets of 5 mg glibenclamide (Tongmei, Togo) were purchased at a local pharmacy in Ouagadougou, capital city of Burkina Faso. Dichloromethane, n-hexane, and butanol were obtained from Carlo-Erba (France). Ethanol and ethyl acetate were procured from Prolabo (France). Silica gel TLC plates F 254 grade were from Macherey-Nagel (Germany).

### 2.2. Equipment

The apparatus used to achieve the bronchorelaxant effect of extracts from leafy stems of *Waltheria indica* includes the following: an isolated organ study device equipped with four organ bath chambers (of 20 mL each), isometric transducers (Emka Technologies, France) for measuring isometric force, an amplifier (Emka Technologies, France) for recording changes in isometric force, and a computer for data acquisition. Other instruments used include water bath (Julabo, GmbH, Germany), analytical balance (Sartorius, Germany), oven (Memmert GmbH, Germany), and rotary evaporator (Buchi, Switzerland).

### 2.3. Plant Material

Leafy stems of *W. indica* were collected from the area of Gomboussougou located in the Centre-South region of Burkina Faso (11 25′ 15, 12 N 0 45′ 32, 4 W). The plant was taxonomically authenticated and a voucher specimen bearing the number ID 16876 was preserved at the herbarium of the Laboratory of Plant Biology and Ecology, University Joseph Ki-Zerbo, Burkina Faso.

### 2.4. Preparation of the Plant Extracts

The leafy stems of *Waltheria indica* were shade-dried under room temperature and powdered with a grinder. Two extracts were prepared from the powdered leafy stems.

The aqueous decoction was obtained by bringing to boil 150 g of grounded leafy stems mixed with 1.5 L of distilled water for 1 h. Then, the extract was filtered and freeze-dried.

To prepare the hydroalcoholic extract, 150 g of powdered leafy stems was macerated in a water and ethanol mixture (1.5 L) in proportion of 20 : 80 (v/v) at room temperature and under mechanical stirring for 48 h. Thereafter, the extract was filtered, concentrated using a rotary evaporator, and lyophilized. The residual moisture's content of the powder and the extraction's yield of the two extracts were determined in accordance with the literature [32, 33].

The two lyophilized extracts were then kept in a freezer until required for the biological assays.

### 2.5. Preliminary Phytochemical Investigation

The powdered leafy stems of *Waltheria indica* as well as the decoction and hydroalcoholic extracts were screened for the presence and/or absence of various secondary metabolites including flavonoids, tannins, alkaloids, saponins, coumarins, steroids, and triterpenoids, using suitable standard qualitative methods [34]. The thin layer chromatography was carried out on precoated plates using two solvent systems: (i) hexane/ethyl acetate/methanol (14/4/2, v/v/v) and (ii) ethyl acetate/methanol/water (20/2, 6/2, v/v/v). Specific spray reagents highlighted the presence of the main phytoconstituents.

### 2.6. Experimental Animals

NMRI strains mice (20–30 g) and Wistar strains rats (300–400 g) of either sex were obtained from the animal facility of the department of Medicine, Traditional Pharmacopeia, and Pharmacy at the Institute of Research in Health Sciences (IRSS). The animals were maintained under standard laboratory conditions (temperature of 22 ± 3°C, 12/12 h light/dark cycle and relative humidity of 50–70%) with ad libitum access to food and water. However, before the experiment, food was drawn back but the animals still had free access to water. Experimental protocols were strictly performed in accordance with the eighth edition of the “Guide for the Care and Use of Laboratory Animals” with minimal number of animal usage according to the 3Rs principle, i.e., Replacement, Reduction, and Refinement [35].

### 2.7. Acute Toxicity Study

To conduct the acute toxicity assay, a limit test at 5000 mg/kg of extract was used in both male and female mice according to the OECD Guidelines 423 [36]. Each animal was treated once with a single oral dose of 5000 mg/kg of extract by gavage using a feeding tube. Prior to the administration of extracts, mice were weighted, marked, and fasted for 3–4 h, but with access to water. Briefly, four groups of three mice each were constituted. The 1^st^ and 2^nd^ groups received orally a single dose of 5000 mg/kg of body weight of aqueous decoction (AD) and hydroalcoholic extract (HE), respectively. The 3^rd^ and 4^th^ were controls groups for AD and HE, respectively; the mice of these groups received distilled water and a 5% aqueous ethanol solution. Two hours after the administration of the extracts, mice were given ad libitum access to food and water. Thereafter, the experiment was repeated with male mice (control and tested groups).

The health condition of the mice was observed each 30 min during the first two hours following the treatment and then, twice a day for 13 days. Mice were monitored to report any possible clinical signs of toxicity including mortality, change in general behavior, movements, and reflexes.

### 2.8. Bronchorelaxant Study

#### 2.8.1. Preparation of Tracheal Rings

The bronchodilation effect of the two extracts from *Waltheria indica* leafy stems was assayed on rat isolated tracheal preparations according to a modified method of Ouedraogo et al. [37]. Rats were starved 24 h prior to starting the experiment and anesthetized with ketamine (1 g/kg of body weight). The animals' chest was opened and the trachea was excised and placed quickly in a Petri-dish containing a modified Krebs-Henseleit physiological solution (having composition (in mM) of NaCl 118; NaHCO_3_ 24.1; KCl 4.7; KH_2_PO_4_ 1.2; MgSO_4_ 2.5; CaCl_2_ 3.33; Glucose 3.33 and the pH was adjusted to 7.4). The trachea was separated from surrounding tissue and cut into four to five rings of about 5 mm in length. Each ring was mounted in a 20 mL organ bath chambers containing a modified Krebs-Henseleit physiological solution, continuously aerated, and maintained at the temperature of 37°C. Tracheal isolated rings were allowed to equilibrate under a tension of 1 g for 1 h, a time during which bathing solution was replaced every 15 min.

#### 2.8.2. Effect of Acetylcholine (ACh) on the Isolated Rat Trachea

Before commencing the experiment, KCl at 80 mM was added in the bath during the plateau phase to assess the contractile reactivity of tracheal rings. Then, the tracheal rings were washed several times with fresh Krebs-Henseleit physiological solution, left to recover, and returned to their initial state. These steps of precontraction with the KCl followed by several washes were continuously repeated before the start-up of each experimental procedure.

In order to determine the concentration of acetylcholine that induces maximal contraction of the tracheal ring, cumulative concentrations of ACh (10^−6^ to 1.5 × 10^−5^ M) were added in the organ bath. A maximal contraction effect was obtained with a concentration of ACh equal to 10^−5^ M.

#### 2.8.3. Effect of *Waltheria indica* Leafy Stem Extracts on the Isolated Rat Trachea

The effects of AD and HE were evaluated on trachea rings precontracted with either ACh 10^−5^ M or KCl 6 × 10^−2^ M, by cumulative addition of the extracts at the concentrations of 10, 30, 100, 300, 1000, 2000, and 3000 *μ*g/mL.

To study whether potassium channels are involved in the mechanism of the bronchorelaxant effect, glibenclamide, an ATP-sensitive potassium channel inhibitor [38], was used. Briefly, glibenclamide at 10^−5^ M was added in the organ bath followed 15 min later with ACh 10^−5^ M. Then, the test extracts were cumulatively added at the respective concentrations of 10, 30, 100, 300, 1000, 2000, and 3000 *μ*g/mL.

### 2.9. Data Presentation and Statistical Analysis

Signs (+) or (-) were used to assess the presence or not of phytochemical groups in the tested extracts. The percentage of relaxation and the EC_50_, i.e., the concentration required to induce 50% of the maximal bronchorelaxant effect, were expressed as mean ± SD (standard deviation) of four experiments (*n* = 4). The EC_50_ values and the plotted curves of the bronchorelaxant effect were obtained with GraphPad Prism^®^ Software (version 6.07). Data were compared by a two-way ANOVA with the Bonferroni correction for multiple comparisons. Differences were considered significant with a *p* value <0.05.

## 3. Results

### 3.1. Preliminary Phytochemical Investigation

The yield of the extracts, the residual moisture content, and the results of the preliminary phytochemical search of *Waltheria indica* leafy stem extracts are summarized in Table 1.

### 3.2. Acute Toxicity Study

The acute toxicity was assessed in mice according to the OECD Guidelines 423. The results showed no mortality at the limit dose of 5000 mg/kg of body weight. Apart from a slight drowsiness observed during the first 30 min following the administration of the plant extracts, there were no significant changes in the general behavior of the extract-treated mice. The administration of the hydroalcoholic extract (HE) decreased mice body weight gain although this was minor. In contrast, the aqueous decoction (AD) caused a slight increase of mice body weight at the end of the experiment period (14 days).

### 3.3. Bronchorelaxant Study

#### 3.3.1. Effect of *Waltheria indica* Leafy Stem Extracts on Acetylcholine- and Potassium chloride- (KCl-) Induced Contractions Response of Isolated Rat Trachea

**Figure 1:**
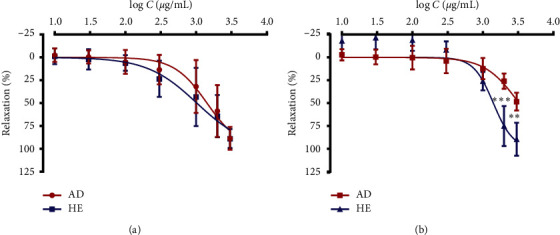
Relaxant effect of the aqueous decoction (AD) and hydroalcoholic extract of *Waltheria indica* leafy stems on acetylcholine (a) and KCl (b) contracted rat tracheal rings. Data and error bars represent the mean ± SD of four independent experiments. Statistical comparisons between the two extracts were assessed using a two-way ANOVA with Bonferroni multiple comparison test. ^*∗∗∗*^*p* < 0.001 and ^*∗∗*^*p* < 0.001 (*n* = 4) indicate the statistical differences.

Figure 1 presents the relaxant effect of *Waltheria indica* leafy stem extracts on acetylcholine- and KCl-precontracted rat tracheal rings (Figures 1(a) and 1(b), respectively). The aqueous decoction and hydroalcoholic extract from the leafy stems of *W*. *indica* dose dependently inhibited the contraction response in tracheal rings contracted by acetylcholine or potassium chloride (KCl). In tracheal rings precontracted with acetylcholine, 10^−5^ M (Figure 1(a)), the aqueous decoction and hydroalcoholic extract elicited an interesting bronchorelaxant response with respective EC_50_ values of 1.517 ± 0.002 mg/mL and 1.200 ± 0.002 mg/mL. The maximal bronchorelaxant effect (Emax) was equal to 88.54 ± 12.44% and 88.86 ± 10.38%, respectively, for the aqueous decoction and the hydroalcoholic extract. There were no statistically significant differences between the two extracts when tested on acetylcholine-induced contractions.

In tracheal rat rings precontracted with KCl 6 × 10^−2^ M (Figure 1(b)), the aqueous decoction and the hydroalcoholic extract produced a maximal bronchorelaxant effect of 48.42 ± 9.71% and 89.53 ± 18.06%, respectively. The hydroalcoholic extract significantly inhibited the KCl-induced contractions and was more potent with an EC_50_ value of 1.433 ± 0.001 mg/mL. The EC_50_ value of the aqueous decoction was slightly higher than 3 mg/mL (the maximum concentration tested) and was equal to 3.159 ± 0.001 mg/mL.

#### 3.3.2. Effect of *Waltheria indica* Leafy Stem Extracts on Acetylcholine-Induced Contractions Response of Isolated Rat Trachea in the Presence of Glibenclamide

**Figure 2:**
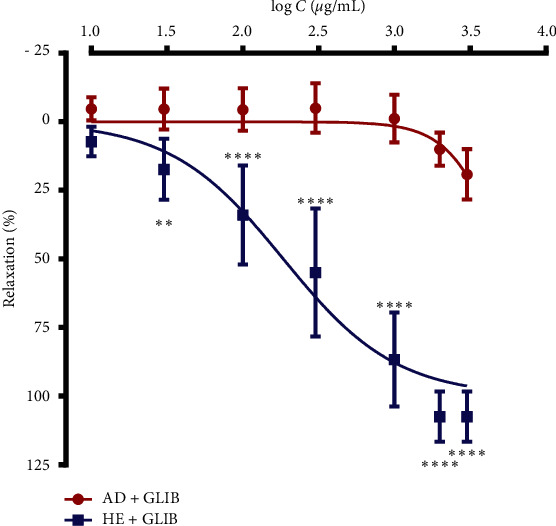
Relaxant effect of the aqueous decoction (AD) and hydroalcoholic extract of *Waltheria indica* leafy stems on acetylcholine-induced contractions in rat tracheal rings in the presence of glibenclamide (GLIB). Data and error bars represent the mean ± SD of four independent experiments. Statistical comparisons between the two extracts were assessed using a two-way ANOVA with Bonferroni multiple comparison test. ^*∗∗∗∗*^*p* < 0.0001 and ^*∗∗*^*p* < 0.01 (*n* = 4) indicate the statistical differences.

Glibenclamide was used to study whether potassium channels are involved in the mechanism of the bronchorelaxant effect of extracts. The tracheal rings were pretreated with glibenclamide at 10^−5^ M before addition of ACh 10^−5^ M in the organ bath 15 min later and then by test extracts.  Figure 2 presents the relaxant effect of *Waltheria indica* leafy stem extracts on acetylcholine-precontracted rat tracheal rings in presence of glibenclamide. The relaxant effect of the aqueous decoction was significantly reduced in presence of glibenclamide. Indeed, in absence of glibenclamide, the pharmacological parameters, namely, the Emax and EC_50_, vary from 88.54 ± 12.44% to 21.09 ± 8.12% for the Emax and from 1.517 ± 0.002 mg/mL to 4.722 ± 0.001 mg/mL for the EC_50_ values. Pretreatment of the tracheal rings with glibenclamide in presence of the hydroalcoholic extract significantly (*p* < 0.0001) increased the Emax (from 88.86 ± 10.38% to 109.88 ± 5.47%) and decreased the EC_50_ value from 1.200 ± 0.002 mg/mL to 0.191 ± 0.002 mg/mL.

## 4. Discussion

*Waltheria indica* L. (Malvaceae) has been claimed to treat various ailments including asthma. This study was undertaken to provide scientific evidence regarding the use of the leafy stems in the management of asthma. The yield of the aqueous decoction extraction was higher than that of the hydroalcoholic extract. This could be due to the effect of heat which contributed to extract more compounds compared to the compounds in the hydroalcoholic extract that were extracted by maceration technique. As recommended, the moisture content was less than 10% suggesting that the powdered leafy stems of *Waltheria indica* are well preserved, without risk of contamination and/or alteration of the phytochemicals [39].

The powdered leafy stems and the hydroalcoholic extract contain alkaloids, flavonoids, tannins, triterpenoids, steroids, coumarins, and saponins. These results corroborate previous studies reviewed by Nirmala and Sridevi [22] and Zongo et al. [23]. However, we noticed the absence of tannins, coumarins, steroids, and triterpenoids in the aqueous decoction. The absence or presence of a particular phytoconstituent in an extract depends on its solubility in the extraction solvent (water/ethanol) or on the extraction method (maceration/decoction) [40]. Consequently, the absence of some secondary metabolites in the aqueous decoction probably means that these substances have been destroyed during the extraction process.

Interestingly, the results of the acute toxicity showed that, up to 5000 mg/kg of body weight, no mortality or any signs of toxicity were observed during the observation period. This result suggests that the extracts were less toxic and therefore the lethal dose (LD_50_) was considered to exceed 5000 mg/kg. The weight loss, though minor observed with the administration of the hydroalcoholic extract at the end of the test period, may be attributed to the presence of tannins and saponins, phytoconstituents known to modulate body weight [41, 42].

In rat tracheal rings precontracted with KCl and acetylcholine, the extracts from the leafy stems of *Waltheria indica* exhibited significant concentration-dependent relaxant effects. The hydroalcoholic extract was more potent at inhibiting the contractions induced by ACh and KCl than the aqueous decoction. These results showed different mechanisms of bronchorelaxant effect. As reported elsewhere [43], contraction of the smooth muscle is mainly linked to the increase of the concentration of intracellular calcium ([Ca^2+^]_*i*_). [Ca^2+^]_*i*_ can originate from extracellular space and intracellular store, especially from the sarcoplasmic reticulum. KCl induced contraction by increasing intracellular calcium ([Ca^2+^]_*i*_) through voltage-dependent Ca^2+^ channels while acetylcholine activates a G-protein-coupled and produces contraction by increasing the Ca^2+^ sensitivity and Ca^2+^ entry via the voltage-dependent Ca^2+^ channels and receptor-operated calcium channels [44]. The receptor-operated calcium channels are coupled to muscarinic receptors and their activation caused the release of Ca^2+^ from the sarcoplasmic reticulum [43, 44]. The ability of hydroalcoholic extract to inhibit the KCl- and ACh-induced contractions suggests that it may decrease Ca^2+^ influx and/or block the receptor-operated calcium channels which in turn affect the release of Ca^2+^ from the sarcoplasmic reticulum. However, the bronchorelaxant effect of the aqueous decoction may be explained by an effect on the receptor-operated calcium channels (ACh-induced contraction) instead of an effect on the voltage-dependent Ca^2+^ channels (KCl-induced contraction).

The extracts were tested on acetylcholine contracted rat trachea preparation in presence of glibenclamide to study whether potassium channels are involved in the mechanism of the bronchorelaxant effect. There are five types of potassium (K^+^) ion channels that play important roles in the regulation of vascular smooth muscle tone. Indeed, their activation produced membrane hyperpolarization leading to vasodilatation, whereas their inhibition caused membrane depolarization and thereby vasoconstriction [45]. In the presence of glibenclamide, an ATP-sensitive potassium channel inhibitor (K^+^-ATP channel), the relaxant effect of the hydroalcoholic extract was higher than in the absence of this inhibitor. This result implies that the hydroalcoholic extract may induce relaxation through different pathways including the decreasing of [Ca^2+^]_*i*_, the blockade of receptor-operated calcium channels, and the opening of K^+^-ATP channels despite the presence of glibenclamide. However, the relaxant effect of the aqueous decoction was significantly reduced in the presence of glibenclamide, suggesting the involvement of the K^+^-ATP channels in its relaxation mechanism.

The major phytoconstituents of the hydroalcoholic extract include alkaloids, flavonoids, tannins, triterpenoids, steroids, coumarins, and saponins. The antiasthmatic properties of most of these phytochemicals have been previously proven and they exert their antiasthmatic effects through bronchodilatation, inhibition of Ca^2+^ channels, and blocking of muscarinic receptors [3, 46, 47]. Zongo et al. [29] have isolated epicatechin from the hydroalcoholic extract of *Waltheria indica* roots. This flavonoid was found to be potent on smooth muscle relaxation and at inhibiting 5-Lox enzyme activity, two important targets for asthma therapy [29]. The relaxant effect of aqueous decoction, though less potent compared to that of hydroalcoholic extract, may be due to the presence of flavonoids, alkaloids, and saponins [3].

## 5. Conclusion

Following the antiasthmatic claim of *Waltheria indica*, the present study was undertaken to study the bronchorelaxant effect of the leafy stems of *Waltheria indica* (Malvaceae). The hydroalcoholic extract stands to be more potent comparatively to the aqueous decoction at inhibiting the contractions induced by both acetylcholine and potassium chloride. The significant effect of the hydroalcoholic extract may be due to the presence of various phytoconstituents which work synergistically through different mechanisms of action to exert the relaxant effect. Furthermore, we hypothesized that the relaxant activity of the aqueous decoction may involve the potassium ion channels, particularly the ATP-sensitive K^+^ channels (K_ATP_). However, further studies are needed to explore other mechanisms of the bronchorelaxant effect and carry out the chemical fingerprinting of the leafy stems of *Waltheria indica*.

## Figures and Tables

**Table 1:** Preliminary phytochemical study results of *Waltheria indica* leafy stem extracts.

Plant part/extract			
Phytochemical compound	Powdered leafy stems	Aqueous decoction	Hydroalcoholic extract
Flavonoids	+	+	+
Saponins	+	+	+
Alkaloids	+	+	+
Tannins	+	−	+
Steroids	+	−	+
Triterpenoids	+	−	+
Coumarins	+	−	+
Residual moisture content (%)	5.83 ± 0.02	n.d.	n.d.
Extract yield (%)		22.21 ± 0.08	14.54 ± 0.02

Abbreviations: (+) present; (−) absent; n.d.: not determined.

## Data Availability

The data used to support the findings of this study are included within the manuscript.
